# Impacts of the Dynamic High-Pressure Pre-Treatment and Post-Treatment of Whey Protein Aggregates on Their Physicochemical Properties and Emulsifying Activities

**DOI:** 10.3390/foods11223588

**Published:** 2022-11-11

**Authors:** Eun Hee Yoo, Chang Woo Kwon, Seung Jun Choi

**Affiliations:** 1 Department of Food Science and Technology, Seoul National University of Science and Technology, Seoul 01811, Korea; 2 Research Institute of Agricultural and Life Science, Seoul National University, Seoul 08826, Korea; 3 Center for Functional Biomaterials, Seoul National University of Science and Technology, Seoul 01811, Korea

**Keywords:** aggregation, dynamic high-pressure treatment, emulsion activity index, thermal treatment, whey protein isolates

## Abstract

The impacts of dynamic high-pressure (DHP) pretreatment and post-treatment (100 MPa) on the physicochemical and functional properties of whey protein isolate (WPI) aggregates formed by thermal treatment were investigated in this study. When WPI aggregates were formed by thermal treatment, the size of the aggregates formed with the DHP pretreated WPI was smaller than that of the aggregates formed with the original WPI. The size of the WPI aggregates formed by thermal treatment decreased with DHP post-treatment. The conformational parameters (ζ-potential, surface hydrophobicity, and intrinsic fluorescence intensity) of the WPI subjected to DHP pretreatment were not significantly influenced by thermal treatment. However, DHP post-treatment affected these parameters for the WPI aggregates formed during thermal treatment because of dissociation caused by intense shear and cavitation forces during DHP treatment. The emulsifying activity index (EAI) of the WPI aggregates slightly improved with DHP treatment, but its order had little effect on the magnitude of the EAI increase. DHP pretreatment or post-treatment can modulate the conformational structures and the physicochemical properties of protein aggregates.

## 1. Introduction

Over the past few decades, nano and microsized particles fabricated from edible biopolymers have attracted increasing attention from researchers due to their possible applications as encapsulation, protection, and delivery systems for bioactive and functional ingredients [1,2,3,4]. Moreover, because the properties of biopolymer particles are similar to those of the oil droplets of some fatty food products present in the mouth, they can potentially serve as fat replacers [5,6].

Among homogeneous and heterogeneous biopolymer particles, protein particles (aggregates) can be fabricated by thermal treatment, which promotes the physicochemical, structural, and functional modifications of protein molecules [7,8]. The inner hydrophobic domains of such molecules are exposed during heating above their thermal denaturation temperature, which enables the formation of protein aggregates through the interactions between these domains [9]. The shapes of protein aggregates, such as particles and fibrils, as well as their sizes are determined by protein solution parameters, including pH, ionic strength, and protein concentration [10,11]. Heating conditions, such as temperature and time, are also important factors affecting the shape and size of protein aggregates. For example, when a protein solution is heated at a pH value close to its isoelectric point (pI), the driving forces for protein aggregation include electrostatic attraction and hydrophobic interactions between denatured protein molecules, resulting in the formation of relatively large protein aggregates. Therefore, the physicochemical properties of protein aggregates formed by heating at a pH value significantly different from pI could be fairly different from those of aggregates formed at a pH value close to its pI. This indicates that protein aggregates with various physicochemical properties (including size and charge) can be prepared by fine-tuning the parameters of the protein solution and heating process.

High-pressure nonthermal processing technology has been used in the food industry to inactivate microorganisms and some proteins, including enzymes [12,13]. Microfluidizer, a widely employed device for dynamic high-pressure (DHP) treatment, forces fluid through microchannels inside its interaction chambers with special configurations (Y- and Z-types and their combination) [14]. During the DHP treatment with a microfluidizer, the very fast movement of a fluid through the microchannels caused by the high pressure induces intense mechanical processes, including turbulence, intense shear, cavitation, and high-velocity impact [14]. DHP treatment is a nonthermal treatment technique; however, the high pressure may cause an increase in temperature [15]. Although the underlying reasons for this phenomenon can affect the tertiary and/or quaternary structures of protein molecules and their functionalities, the changes in the molecular protein conformation induced by high-pressure treatment may differ from those caused by thermal treatment. However, proteins are very likely to undergo different processes during the DHP treatment inside a microfluidizer as compared with those induced by a static high-pressure treatment. The stream flowing through the microchannels of the microfluidizer interaction chamber at a large speed due to high pressure reaches the impingement area [16]. Therefore, extreme shear, impact, and cavitation forces are generated in the interaction chamber [17], while the physicochemical properties of the protein aggregates produced by denaturation during DHP treatment can differ from those of the protein aggregates formed by either static high-pressure or thermal treatment. The effect of the high-pressure treatment of whey proteins and soy proteins on their emulsifying properties has been investigated previously [18,19]. The obtained results revealed that the DHP treatment of heated whey and soy proteins increased their emulsifying activity index (EAI). The observed effect of DHP treatment on the surface properties of thermal pea albumin aggregates showed that high-pressure treatment had little impact on the foaming properties of these aggregates at low pH but considerably enhanced them at the neutral pH [20]. DHP treatment has also been used to increase the solubilities of heated whey proteins [21] and insoluble pea proteins [22]. These physicochemical changes were likely caused by the reduced size and increased flexibility of protein aggregates induced by DHP treatment [20]. Thus, high-pressure treatment strongly influences the functionalities of protein aggregates. By successively performing thermal and high-pressure treatment for a protein solution, it is possible to fabricate protein aggregates with different physicochemical properties as compared with those of the aggregates formed during thermal treatment or high-pressure treatment only.

Therefore, the objective of this study was to investigate the impacts of DHP treatment on the physicochemical properties and functionalities of protein aggregates formed by thermal treatment. For this aim, whey protein isolates (WPIs), the byproducts of cheese making, were selected because they are incorporated into different types of food products due to their various functionalities, such as foaming, emulsifying, and gelling capabilities [23,24]. As noted above, since DHP treatment can impact the molecular structures of WPIs, WPI aggregates prepared by thermal treatment after DHP treatment may have significantly different physicochemical and functional properties from those obtained by thermal treatment alone. In the same manner, since DHP treatment can affect the molecular structures of WPI aggregates formed by thermal treatment, their physicochemical and functional properties may also differ from those obtained by thermal treatment alone. In this study, WPI aggregates were formed during thermal treatment, DHP treatment, or their combinations at pH values below, above, and close to the pI of WPI molecules, and their size, charge, surface hydrophobicity, and emulsifying properties were determined.

## 2. Materials and Methods

### 2.1. The Materials

WPI 9400 was kindly donated by Hilmar Ingredients (Hilmar, CA, USA) and used directly from the sample pouch without further purification. According to the manufacturer specifications, it contained 93.4% of protein and 2.6% of ash. 1-Anilino-8-naphtalenesulfonate (ANS) was purchased from Alfa Aesar (Ward Hill, MA, USA). Soybean oil was obtained from a local supermarket. All other chemicals of analytical grade were purchased from Sigma-Aldrich (St. Louis, MO, USA), Fisher Scientific (Fair Lawn, NJ, USA), Daejung Chemicals and Metals (Siheung, Korea), and Duksan General Science (Ansan, Korea).

### 2.2. Preparation of a WPI Solution

WPI solution (1% (*w/v*)) was prepared by dissolving WPI powder with a 200 mL acetate buffer (10 mM, pH 7) in a 250 mL glass bottle, and sodium azide (0.02% (*w*/*v*)) was added into the WPI solution to prevent the growth of microorganisms. The obtained WPI solution was continuously stirred at ambient temperature at a speed of 150 rpm for 8 h, after which its pH was adjusted to 7.0 using 1.0 N sodium hydroxide and hydrochloric acid solutions.

### 2.3. Preparation of WPI Aggregates

The WPI solution was subjected to thermal treatment to produce particles in the sub-micrometer range. For this purpose, the freshly prepared WPI solution was heated to 85 °C above its denaturation temperature for 15 min in a water bath. After thermal treatment, the WPI solution was cooled to 25 °C using a water bath and kept at that temperature for 2 h. Subsequently, the pH of the WPI solution was adjusted to the desired value using 1.0 N hydrochloric acid solution and then stirred for at least 30 min before the next step. DHP treatment was conducted by subjecting WPI solution to 1 pass through a microfluidizer (MN400BF, Micronox, Seongnam, Korea) equipped with a Z-type interaction chamber at a pressure of 100 MPa. In order to investigate the effect of DHP treatment on the characteristics of WPI aggregates, the DHP treatment procedure was performed before and after the thermal treatment step.

*DHP pretreatment*: the WPI solution was subjected to the DHP treatment at pH = 7. The pH of the resultant solution was adjusted to 3, 5, or 7, followed by thermal treatment. The obtained samples were labeled P7T3, P7T5, and P7T7, respectively.

*DHP post-treatment*: the thermal treatment of the WPI solution was conducted at pH = 7. The pH of the thermally-treated WPI solution was adjusted to 3, 5, or 7, followed by DHP treatment. The obtained samples were labeled T7P3, T7P5, and T7P7, respectively.

### 2.4. Particle Characterization of WPI Aggregates

A dynamic light scattering apparatus (SZ-100; Horiba, Kyoto, Japan) was used to determine the particle size, polydispersity index, and surface charge of the WPI aggregates. The aggregate size data were reported as Z-average mean diameters, and the aggregate charge data were reported as ζ-potentials. All measurements were performed at 25 °C.

### 2.5. Fluorescence Spectroscopic Analysis of WPI Aggregates

Surface hydrophobicity changes were determined according to the method of Cao, Zhao, and Xiong (2016) [25]. The WPI aggregate solution was diluted to 0.05, 0.10, 0.15, 0.20, and 0.25 mg/mL in a 10 mM acetate buffer having the same pH to WPI solution. Afterward, 20 μL of 8.0 mM ANS (dissolved in 100 mM phosphate buffer (pH 7)) was added to 4.0 mL of the diluted WPI aggregate solution and mixed vigorously. Fluorescence intensity was measured exactly 15 min after ANS addition using a fluorescence spectrophotometer (SpectraMax i3x, Molecular Devices, San Jose, CA, USA) with excitation and emission wavelengths of 390 and 470 nm, respectively. The fluorescence intensity values of the sample blank (WPI aggregate solution without ANS) and reagent blank (sodium acetate buffer without WPI aggregates but with ANS) were measured and subtracted from those of the samples (WPI aggregate solution with ANS). The surface hydrophobicity (*H*_0_) of the WPI aggregates was expressed as the initial slope of the fluorescence intensity versus the WPI concentration plot calculated by linear regression analysis.

Intrinsic fluorescence changes were determined by a fluorescence spectrophotometer (SpectraMax i3x, Molecular Devices, San Jose, CA, USA). The WPI aggregate solution was diluted to 0.5 mg/mL using 10 mM acetate buffer having the same pH as the WPI solution. The excitation wavelength was set to 290 nm, and emission spectra were recorded in a wavelength range from 310 to 500 nm at a slit width of 1 nm.

### 2.6. Characterization of Emulsions Containing WPI Aggregates

A coarse emulsion was prepared by mixing 5% (*w*/*w*) of the oil phase (soybean oil) with 95% (*w*/*w*) of the aqueous phase (WPI sample solution) using a high-shear mixer for 2 min at 25 °C. A coarse emulsion was homogenized by conducting five passes at 100 MPa using a microfluidizer (MN400BF, Micronox, Seongnam, Korea). The emulsifying activity index (EAI) value was determined according to the method developed by Pearce and Kinsella (1978) because it provided an estimate of the relative surface coverage of a protein on an oil droplet within a dilute emulsion [26]. Immediately after emulsion fabrication, a 50 μL aliquot was added to 5 mL of the pH-adjusted aqueous solution containing 0.1% (*w*/*w*) sodium dodecyl sulfate and vortexed for 10 s. Subsequently, the diluted emulsion was transferred into a plastic cuvette (path length: 1 cm), and its absorbance at 500 nm was measured with an Optizen Pop spectrophotometer (Mecasys, Daejeon, Korea). The EAI was calculated via the following equation:

EAI m2g=2·2.303·A0·DFc·ϕ·10000

where $A_{0}$ is the absorbance of the diluted emulsion measured immediately after fabrication, DF is the dilution factor (100×), c is the protein weight per unit volume (g/mL), and ϕ is the oil volume fraction of the emulsion.

### 2.7. Statistical Analysis

All experiments were conducted in triplicate using freshly prepared samples and the data are expressed as mean ± standard deviation. An analysis of variance (ANOVA) and Duncan’s multiple-range test (*p* ≤ 0.05) were conducted using SPSS software version 26.0 (IBM, Armonk, NY, USA).

## 3. Results and Discussion

### 3.1. Influence of DHP Treatment on WPI Physicochemical Characteristics

The particle size of native WPI fully hydrated at pH 7 was 165.3 nm (Table 1). β-Lactoglobulin and α-lactalbumin, with relative contents of approximately 50% and 20%, were the major whey proteins. β-Lactoglobulin is about 3.6 nm in diameter, and it exists as a monomer, dimer, or tetramer depending on pH [27], and α-lactalbumin is very similar in size to β-lactoglobulin [28]. However, the particle size of the native WPI obtained in this study was much greater than the sizes of β-lactoglobulin and α-lactalbumin. Because commercial WPI products are typically produced through thermal and spray-drying processes, their WPI components are often denatured and/or aggregated. Therefore, the particle size of the WPIs obtained in this study represents the size of the WPI aggregates rather than the size of individual WPI proteins. *H*_0_ can be varied by changing the spatial structure of proteins, especially by exposing the hydrophobic domains buried within a protein’s structure to its surface [29]. However, the much higher *H*_0_ value of the original (or native) WPI aggregates obtained at pH 3, rather than at pH 7, did not indicate that a larger number of hydrophobic sites were exposed to the WPI surface at the former pH value. The *H*_0_ magnitude determined by the ANS anionic probe at an acidic pH is generally overestimated as compared with that at a neutral pH because ANS can interact with positively charged amino acid residues on the protein’s surface [30]. With an increase in the hydrophilicity of the local microenvironment of amino acids containing aromatic residues (such as tryptophan), an increase in the intrinsic fluorescence intensity (*I*_max_) and a shift of the maximum fluorescence emission wavelength (*λ*_max_) to larger values are commonly observed [31]. The change in pH did not change the *I*_max_ of β-lactoglobulin, the major whey protein [32]; however, in the present study, the *I*_max_ of the WPI increased with decreasing pH (Table 1). Because a change in pH affects weak interactions (such as van der Waals and electrostatic interactions, which effectively stabilize the original WPI aggregates), the hydrophobic domains buried within these aggregates can be exposed. This is one of the likely reasons for the observed change in the *H*_0_ and *I*_max_ of the original WPI aggregates with pH variation.

As described above, because the WPIs are subjected to a very harsh environment during DHP treatment, considerable changes in the physicochemical properties of the original WPI aggregates may have occurred due to the denaturation of the individual proteins (β-lactoglobulin, α-lactalbumin, and minor proteins) that make up the WPI aggregates and/or the alteration of the conformational structures of the WPI aggregates themselves. However, although the DHP treatment at pH 3 slightly decreased the size of the original WPI aggregates and the DHP treatment at pH 7 slightly increased the size of the WPI aggregates, the values for ζ-potential, *H*_0_, *I*_max_, and *λ*_max_ remained intact in most cases (Table 1 and Table 2). According to the results of previous studies [33,34], DHP treatment decreased the particle size of insoluble whey protein aggregates and increased their *H*_0_ magnitude due to the deaggregation (or disassociation) of these aggregates by mechanical forces. Therefore, two possible explanations for the observed change in the WPI particle size after DHP treatment were suggested. First, it could result from the partial disassociation of the original WPI aggregates into smaller aggregates without a significant change in the overall conformational configuration. Second, the original WPI aggregates were disassociated into their subunits, which were subsequently reassociated into aggregates with different sizes. However, the very small changes in *H*_0_, *I*_max_, and *λ*_max_ indicated that the conformation structure of the original WPI aggregates was not affected by DHP treatment and that it was practically impossible for the reassembled aggregates to retain the same conformational structure of the original aggregates. The results of a previous study revealed that the tertiary structure of proteins subjected to high-pressure treatment could be rearranged without a significant change in their overall conformational configuration due to the partial change in the molecular volume of globular proteins [35]. Therefore, it could be concluded that the different particle sizes of the WPI aggregates in the Px samples (WPI samples subjected to the DHP treatment at pH x), when compared with those of the original WPI aggregates, could be due to the change in their molecular volume at the same overall conformational (tertiary and/or quaternary) configuration during DHP treatment. Previous works revealed that DHP treatment conducted at 100 MPa changed the contents of secondary structures such as α-helix, β-sheet, and random protein coils [36,37], which likely triggered the conformational changes of the proteins. Therefore, the WPI aggregates adopted a stable conformational state because the alteration of the secondary structures of the subunits within the WPI aggregates promoted the WPI molecular rearrangement. However, in this study, the contents of α-helix (15.6%), β-sheet (36.9%), and random coil (31.4%) in the native WPI rarely changed after DHP treatment.

In order to evaluate the effect of DHP treatment on the physicochemical properties of WPIs, the physicochemical properties of the Px samples were compared with those of the respective Tx samples (WPI samples thermally treated at pH x). As shown in Table 1 and Table 3, the particle size and *H*_0_ of the WPIs significantly increased after thermal treatment regardless of the pH of the WPI solution, indicating the occurrence of dramatic changes in the conformational structure of the original WPI aggregates. This phenomenon can be attributed to the exposure of the inner hydrophobic domains initially buried inside the original WPI aggregates to their surfaces. The increased *I*_max_ after thermal treatment provided additional evidence for the exposure of tryptophan originally buried inside the inner hydrophobic cores. Because the state (in which the hydrophobic domains are exposed to water) is thermodynamically unstable, WPI aggregates that are larger than the original WPI aggregates can be formed after thermal treatment due to the strong interactions between the exposed hydrophobic domains [38,39]. The most significant change in the conformational structure of the original WPI aggregates during thermal treatment was observed at pH 5. The morphologies of the WPI aggregates thermally formed at a pH close to their pI may be significantly different from those of aggregates formed by the thermal treatment at a pH far from the pI of the WPI because electrostatic interaction contributed to WPI aggregation as strongly as the hydrophobic interaction [40].

The particle size of the T5 sample remained large after changing its pH to 3 (1322.5 nm) and 7 (7024.2 nm); however, the same pH changes decreased the particle size of the P5 sample to 208.9 and 205.1 nm, respectively. Nevertheless, the particle sizes of the P5 sample after the pH adjustments to 3 and 7 were greater than those of the P3 and P7 samples. Therefore, although the particle size increase due to hydrophobic interactions cannot be neglected, these interactions (which represent the main driving force for the formation of WPI aggregates during thermal treatment) may not be the main reason for the slight change in the size of the WPI aggregates observed after DHP treatment. The latter phenomenon was likely caused by the rearrangement of the conformational configuration of the original WPI aggregates induced by the changes in their molecular volume.

### 3.2. Influence of DHP Pretreatment on the Physicochemical Characteristics of WPI Aggregates

In order to evaluate the effect of DHP pretreatment on the physicochemical properties of the WPI aggregates, P7Tx samples (WPI samples pretreated by DHP treatment at pH 7 and thermally treated at pH x) were fabricated by the thermal treatment of the P7 sample (Table 4).

The particle size of the P7T7 sample was smaller than that of the T7 sample and, interestingly, slightly smaller than that of the P7 sample. The particle size of the P7T3 sample was slightly smaller than that (188.9 ± 1.5 nm) of the P7 sample (simply adjusted to pH 3), and the particle size of the P7T3 sample was also smaller than that of the T3 sample. The particle size of the P7T5 sample was similar to those of the T5 and P7 samples, with the pH changed to 5 (3463.3 ± 111.6 nm). The change in pH of the P7T5 sample did not cause the dissociation of the WPI aggregates, indicating that the WPI aggregates in the P7T5 sample were formed by both hydrophobic interaction and electrostatic attraction (or the weak electrostatic repulsion), and that the hydrophobic interaction would be more important in their formation.

The ζ-potential of the P7T7 sample was not significantly different from those of the P7 and T7 samples (the same results were obtained for the P7T7 and P7T5 samples). When the pH of the P7 sample was adjusted to 3, its ζ-potential was 12.46 ± 0.15 mV; this value was very close to the ζ-potentials of the P3 and T3 samples (Table 1 and Table 2). However, the ζ-potential of the P7T3 sample was lower than that of the P7 sample adjusted to pH 3 and the T3 samples. This suggests that the number of amino acids with negatively charged residues on the surface of the WPI aggregates in the P7T3 sample was higher than that in the T3 sample and the P7 sample with a pH adjusted to 3, or that the number of amino acids with positively charged residues on the surface of the WPI aggregates in the P7T3 sample was lower than that in the T3 sample and the P7 sample with pH adjusted to 3. Note that finding a plausible explanation for this phenomenon was a challenging task. The hydrolysis of glutamine and/or asparagine into its acidic forms at highly acidic pH and high-temperature values is one of the possible reaction mechanisms [41]. However, this hypothesis cannot explain the same ζ-potentials of the original WPI aggregates (at pH 3) and the WPI aggregates in the T3 sample. Although the underlying mechanism for this phenomenon is not yet fully understood, it might be closely related to the conformational rearrangement of the WPI aggregates caused by DHP pretreatment.

The *H*_0_ of the P7T7 sample was similar to that of the T7 sample but higher than the *H*_0_ of the P7 sample. The *H*_0_ of the P7T3 sample exceeded that of the P7 sample with the pH adjusted to 3 ((97.34 ± 1.25) × 10^6^), and the *H*_0_ of the P7T5 sample was much higher than that of the P7 sample with the pH changed to 5 ((20.55 ± 0.04) × 10^6^). These results indicate that thermal treatment dramatically increased the *H*_0_ of the P7 sample regardless of the pH level. Hence, a significant conformational change caused by exposing the hydrophobic domains buried inside the WPI aggregates in the P7 sample to their surface occurred during thermal treatment. However, the *H*_0_ of the P7T5 sample was lower than that of the T5 sample, and the *H*_0_ of the P7T3 sample was higher than that of the T3 sample, which suggests that the conformational structures of the P7T3 and P7T5 samples were considerably different from those of the T3 and T5 samples, respectively.

Comparing the *I*_max_ and *λ*_max_ values of the P7 sample with the pH adjusted to 3 ((65.04 ± 2.13) × 10^6^ and 338.8 ± 1.2 nm, respectively), only the *I*_max_ of the P7 sample increased due to thermal treatment at pH 3. However, the *I*_max_ and *λ*_max_ values of the P7T7 sample were much greater than those of the P7 sample but not significantly different from the *I*_max_ and *λ*_max_ magnitudes of the T7 sample. Moreover, thermal treatment dramatically increased the *I*_max_ and *λ*_max_ values of the P7 sample at pH 5 ((54.27 ± 1.04) × 10^6^ and 337.8 ± 0.2 nm, respectively) because the pH adjustment to 5 (close to the pI of the WPIs) reduced the electrostatic repulsion between the protein molecules. This indicates that the formation and growth of the WPI aggregates occurred mainly due to the hydrophobic interaction between the WPI aggregates during the thermal treatment at pH 5. Therefore, some hydrophobic sites on the molecular surface could not participate in the hydrophobic interaction, which was a possible reason for the higher *H*_0_, *I*_max_, and *λ*_max_ values of the P7T5 sample when compared with those of the P7T7 and P7T3 samples. By analyzing the *H*_0_, *I*_max_, and *λ*_max_ magnitudes of the P7Tx samples, it was concluded that a considerable number of hydrophobic sites were exposed to the surface of the WPI aggregates during thermal treatment.

Therefore, the changes in the physicochemical properties of the WPI aggregates in the P7 sample that occurred during thermal treatment considerably differed from the changes observed for the original WPI aggregates. Furthermore, the physicochemical properties of the WPI aggregates in the P5 sample were strongly affected by the pH of the reaction medium.

### 3.3. Influence of DHP Post-Treatment on the Physicochemical Characteristics of the WPI Aggregates

In order to evaluate the effect of DHP post-treatment on the physicochemical properties of the WPI aggregates, DHP treatment was performed on the WPI aggregates fabricated by thermal treatment at pH 7 (T7 sample) (Table 5).

The particle sizes of the WPI aggregates in the T7Px samples (WPI samples (thermally treated at pH 7) subjected to the DHP treatment at pH x) were significantly lower than that of the WPI aggregates in the T7 sample, which likely resulted from their dissociation by mechanical forces during DHP post-treatment. Small WPI aggregates produced by dissociation during DHP post-treatment can exist in the system as they are. When the contact area between the hydrophobic sites and water molecules increases due to the dissociation of the WPI aggregates, the broken WPI aggregates partially reaggregate by hydrophobic interaction or rearrangement. Interestingly, the size of the WPI aggregates in the T7 sample was dramatically reduced by DHP treatment, while the size of the original WPI aggregates changed only slightly after the same treatment. Previous studies revealed that DHP treatment broke large protein aggregates into smaller aggregates [20,33,42]; in particular, the DHP treatment performed at 160 MPa broke large whey protein aggregates into aggregates with sizes of approximately 200 nm [33]. When colloidal aggregates were exposed to a shear flow, the breakage rate of the aggregates with a higher radius of gyration was higher than that of the aggregates with a smaller radius of gyration [43]. Therefore, the WPI aggregates in the T7 sample with a mean particle diameter of 236.5 nm might be more susceptible to mechanical stress during DHP treatment than the original WPI aggregates with a mean particle diameter of 165.3 nm. This suggests that the typical size of the WPI aggregates broken under DHP treatment conditions (100 MPa) was approximately 200 nm. Because the particle size of the original WPI aggregates was relatively small (165.3 nm), they did not disassociate during DHP treatment and retained their original conformational structure with a small size change. This can be a plausible explanation for the very little change in the particle size of the original WPI aggregates after DHP treatment.

The absolute ζ-potential of the T7P7 sample was lower than that of the T7 sample but not significantly different from the absolute ζ-potential of the P7 sample (Table 2, Table 3 and Table 4). The ζ-potential of the T7P3 sample was smaller than that of the T7 sample adjusted to pH 3 (13.38 ± 0.79 mV) and the P3 sample (Table 2, Table 3 and Table 5). Owing to the dissociation of the WPI aggregates by DHP post-treatment, the amino acids with charged residues buried within the WPI aggregates were exposed to the aggregate surface. For this reason, the magnitude of the ζ-potential values of the T7P3 and T7P7 samples decreased after DHP post-treatment. After the dissociation during DHP post-treatment, the WPI aggregates underwent intra and/or intermolecular rearrangement, reducing the contact area between the hydrophobic domains on their surfaces and the water molecules. The movement of the amino acids with charged residues on the aggregate surfaces can also be a possible reason for the decreased absolute ζ-potential values of the T7P3 and T7P7 samples.

The DHP post-treatment of the T7 sample at pH 7 increased the *H*_0_ of the T7 sample, and the *H*_0_ value of the T7 sample subjected to DHP post-treatment at pH 3 was higher than that of the T7 sample adjusted to pH 3 ((197.58 ± 5.13) × 10^6^). However, the *H*_0_ of the T7 sample with a pH adjusted to 5 ((89.05 ± 0.48) × 10^6^) exceeded that of the sample subjected to DHP post-treatment at pH 5. Meanwhile, the DHP post-treatment of the T7 sample at pH 7 did not affect the *I*_max_ and *λ*_max_ values of its WPI aggregates, and the DHP post-treatment of the T7 sample at pH 3 did not change its *I*_max_ magnitude. However, the *I*_max_ and *λ*_max_ values of the T7P5 sample were higher than those of the T7 sample with the pH adjusted to 5. As described above, because mechanical forces can dissociate the large WPI aggregates in the T7 sample during DHP treatment, the observed increase in the *H*_0_ of the T7 sample was due to the larger number of hydrophobic domains exposed to the aggregate surface. The similar *I*_max_ and *λ*_max_ magnitudes obtained after DHP post-treatment indicated that the microenvironment surrounding tryptophan residues was not apparently affected by this process. As indicated by the obtained *H*_0_ values, although the conformational structure of the WPI aggregates was highly influenced by the DHP post-treatment, the amounts of tryptophan residues within the hydrophobic cores that were exposed to the aqueous phase did not change. However, because variations in the *H*_0_ and *I*_max_ values of the proteins are good indicators of changes in their conformational structures, the observed discrepancy between the *H*_0_ and *I*_max_ trends should be examined in more detail.

### 3.4. Emulsifying Properties of the WPI Aggregates

During emulsification, the proteins are first adsorbed at the water–oil interface and then undergo conformational changes to minimize the number of contacts between their hydrophobic groups and the water molecules [44]. These changes mainly involve the unfolding of protein chains to expose the hydrophobic amino acids originally located in the hydrophobic interior of the proteins. This indicates that the proteins with more rigid structures rearrange more slowly than those with less rigid or flexible structures. As described above, because the WPI aggregates fabricated in this study had different structures, they exhibited different rearrangement rates at the water–oil interface. In order to investigate the effects of DHP pretreatment and post-treatment on the emulsifying properties of WPI aggregates, the EAI values of their respective emulsions were determined. The electrostatic repulsion between protein molecules that are adsorbed on the oil droplet surface is the main mechanism for preventing droplet flocculation [45]. It indicates that emulsions stabilized with proteins tend to flocculate at pH values close to the isoelectric point (pI) of the adsorbed proteins. According to the preliminary study, the pI of the WPI used in this study was approximately 4.5. Because pH 5 is close to the pI of WPI, the emulsions fabricated at pH 5 were very sensitive to droplet flocculation. It was not possible to determine the EAI of the WPI aggregates at pH 5. Therefore, the P5, T5, P7T5, and T7P5 samples were excluded from EAI determination.

The droplet size of the emulsion prepared from native WPIs at pH 3 was approximately 0.433 μm (*d*_43_), and the emulsions fabricated from the P3, T3, P7T3, and T7P3 samples demonstrated significantly smaller droplet sizes (approximately 0.422 μm) than those produced from the native WPIs, indicating that the order of the DHP treatment did not affect the emulsion droplet size. The droplet size of the emulsion fabricated from the native WPIs at pH 7 was approximately 0.381 μm (*d*_43_). The droplet sizes of the emulsions prepared from the P7, T7, P7T7, and T7P7 samples were similar to those of the emulsions fabricated from native WPIs. As described above, when the proteins adsorbed at the water–oil interface unfold, the exposed hydrophobic amino acids can interact with the exposed hydrophobic amino acids of the neighboring WPI molecules through hydrophobic attraction. Finally, a viscoelastic interface can be created through the hydrophobic interaction between the different proteins adsorbed at the interface [46]. According to Table 1, Table 2, Table 3 and Table 4, the WPIs aggregated after the treatment at pH 3 exhibited higher *H*_0_ and *I*_max_ values than those at pH 7. As described earlier, *H*_0_ can be overestimated at acidic pH values; hence, if only the *I*_max_ of the WPI aggregates was determined, the number of exposed hydrophobic amino acids (including tryptophan) in the WPI aggregates treated at pH 3 would be higher than in those treated at pH 7. Therefore, after unfolding at the water–oil interface during homogenization, the WPI aggregates treated at pH 3 interacted with the neighboring molecules faster than the WPI aggregates treated at pH 7. Because the more viscoelastic interface at an acidic pH more actively resisted droplet breakage during homogenization, the emulsions fabricated at pH 7 exhibited smaller droplet sizes than those of the emulsions produced at pH 3.

**Figure 1:**
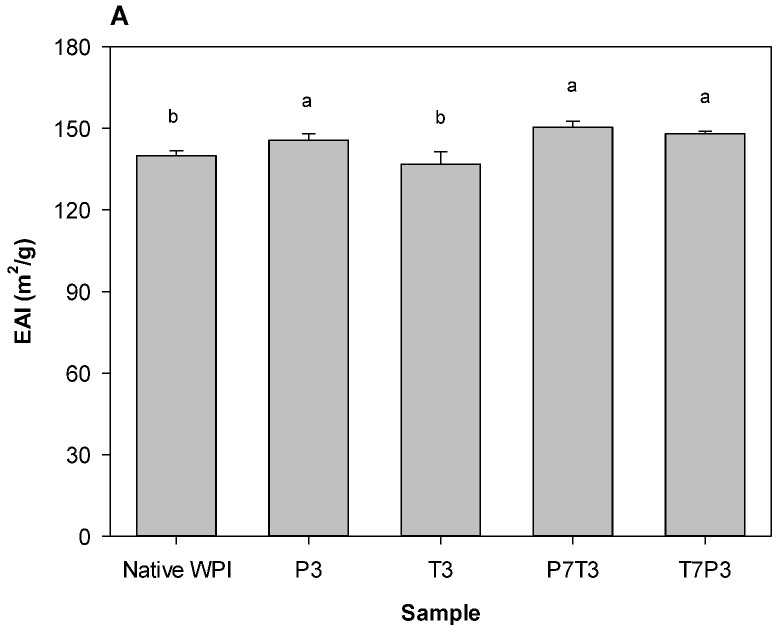

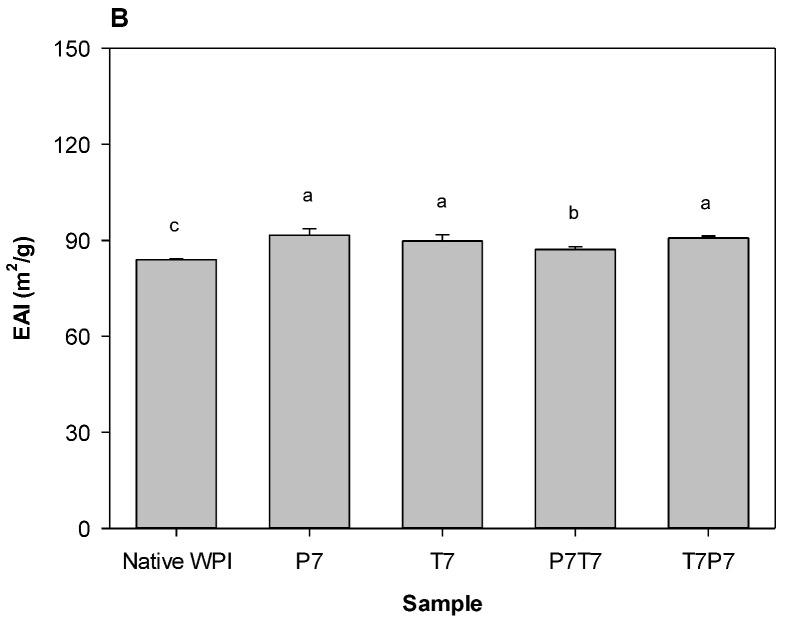
Emulsifying activity indexes of the WPI aggregates subjected to DHP treatment. (**A**) pH 3, (**B**) pH 7. Means denoted by different letters indicate significant differences between samples.

Although DHP treatment increased the EAI of the WPIs regardless of its ordering, the observed EAI increase was significant but small (Figure 1). At pH 3 (emulsions fabricated with P3, T3, P7T3, or T7P3 sample), the emulsions prepared from the WPIs that underwent DHP treatment in any order demonstrated slightly higher EAI values than those of the emulsions produced from the native WPI, and the EAI values of the emulsions prepared from the P3, P7P3, and T7P3 samples were similar to each other. Interestingly, the EAI of the emulsion fabricated from the T3 sample was not different from that of the emulsion prepared from native WPIs. The emulsions fabricated at pH 7 (emulsions fabricated with P7, T7, P7T7, or T7P7 sample) exhibited higher EAI values than those of the emulsions prepared from the native WPI regardless of the treatment type and DHP treatment order. According to the results of a previous study [19], the proteins with higher *H*_0_ values generally possess higher EAI magnitudes. However, although the *H*_0_ values of the P3 and P7 samples were very close to that of the native WPI, their EAI values were greater than the EAI of the native WPI. However, it was found previously that the EAI values of proteins are not correlated with their surface hydrophobicity [47]. This suggests that other factors besides *H*_0_ may affect the emulsifying properties of WPI aggregates. In particular, the effect of DHP treatment on the conformational stability and flexibility of WPIs must be considered [19,20]. Although the physicochemical properties of the P3 and P7 samples (including *H*_0_ and *I*_max_) were not different from those of the native WPI, the WPI flexibility in these samples was higher than that in the native WPI. Therefore, after adsorption at the oil–water interface, the WPI in the P3 and P7 samples unfolded more easily than the native WPIs.

During storage, phase separation proceeded within all emulsions, and a creamed layer on the top of the emulsions was observed after storage for 28 days. This indicated that all emulsions stabilized with the WPI aggregates, regardless of the DHP treatment order and pH, and were not stable under gravitational separation. However, the creamed layer on the top of the emulsions was redispersed easily by gentle shaking. In addition, the size of the droplets in the emulsions stored for 28 days was not significantly different from the size of the droplets in the freshly prepared emulsions, indicating that the origin of the creamed layer was droplet flocculation rather than droplet coalescence and that the structural integrity of the emulsion droplets was maintained during storage.

## 4. Conclusions

This study demonstrated that both DHP pretreatment and post-treatment noticeably affected the conformational structures of WPI aggregates formed by thermal treatment. Although DHP treatment reduced the size of the original WPI aggregates at pH 3 and 7, no significant changes in their ζ-potential, *H*_0_, and *I*_max_ values were observed. DHP pretreatment prevented the size increase of the WPI aggregates during thermal treatment regardless of the pH condition for thermal treatment. However, the conformational structure of WPI subjected to DHP pretreatment resisted thermally-induced denaturation and aggregation differently with changes in pH for thermal treatment. DHP post-treatment reduced the size of the WPI aggregates formed during thermal treatment. The structural change, particularly *H*_0_, of the WPI aggregates formed by thermal treatment was more pronounced in DHP post-treatment at pH 3 than in the DHP treatment at pH 7. DHP treatment slightly increased the EAI of the WPIs regardless of the DHP treatment order. These findings suggest that DHP pretreatment or post-treatment can be performed to modulate the conformational structures of protein aggregates and adjust their physicochemical properties. Therefore, in future studies, DHP treatment conditions (such as pressure, pass number, and temperature) should be optimized for the production of WPI aggregates with desirable physicochemical characteristics.

## Figures and Tables

**Table 1:** The mean particle size, ζ-potential, surface hydrophobicity (*H*_0_), intrinsic fluorescence intensity (*I*_max_), and maximum fluorescence emission wavelength (*λ*_max_) of native WPI.

	pH of Sample		
	3	5	7
Mean particle diameter (nm)	183.05 ± 1.72 ^b^	3626.15 ± 362.06 ^a^	165.3 ± 3.7 ^b^
ζ-potential (mV)	11.17 ± 0.62 ^a^	−5.03 ± 0.77 ^b^	−20.27 ± 1.00 ^c^
*H*_0_ (×10^6^)	92.38 ± 0.39 ^a^	21.63 ± 0.75 ^b^	15.92 ± 0.32 ^c^
*I*_max_ (×10^6^)	62.74 ± 1.26 ^a^	54.15 ± 0.78 ^b^	49.16 ± 1.73 ^c^
*λ*_max_ (nm)	338.7 ± 1.4 ^a^	338.3 ± 0.0 ^a^	337.5 ± 0.2 ^a^

The values with different superscripts in the same row are significantly different as per Duncan’s multiple range test (*p* ≤ 0.05).

**Table 2:** The effect of dynamic high-pressure treatment on the mean particle size, ζ-potential, surface hydrophobicity (*H*_0_), intrinsic fluorescence intensity (*I*_max_), and maximum fluorescence emission wavelength (*λ*_max_) of WPI.

	Sample		
	P3	P5	P7
Mean particle diameter (nm)	176.5 ± 1.3 ^b^	4392.8 ± 281.1 ^a^	184.6 ± 1.1 ^b^
ζ-potential (mV)	12.42 ± 0.28 ^a^	−4.03 ± 0.30 ^b^	−21.36 ± 0.66 ^c^
*H*_0_ (×10^6^)	94.11 ± 1.22 ^a^	21.53 ± 1.07 ^b^	15.92 ± 0.32 ^c^
*I*_max_ (×10^6^)	63.80 ± 0.56 ^a^	53.53 ± 0.25 ^b^	49.11 ± 0.79 ^c^
*λ*_max_ (nm)	338.7 ± 0.5 ^a^	338.2 ± 0.2 ^a^	337.8 ± 0.7 ^a^

The values with different superscripts in the same row are significantly different as per Duncan’s multiple range test (*p* ≤ 0.05).

**Table 3:** The effect of thermal treatment on the mean particle size, ζ-potential, surface hydrophobicity (*H*_0_), intrinsic fluorescence intensity (*I*_max_), and maximum fluorescence emission wavelength (*λ*_max_) of WPI.

	Sample		
	T3	T5	T7
Mean particle diameter (nm)	214.5 ± 0.9 ^b^	5384.7 ± 1007.3 ^a^	236.5 ± 0.6 ^b^
ζ-potential (mV)	12.18 ± 1.11 ^a^	−4.62 ± 0.69 ^b^	−21.99 ± 0.67 ^c^
*H*_0_ (×10^6^)	98.93 ± 5.79 ^b^	272.33 ± 0.32 ^a^	19.82 ± 0.07 ^c^
*I*_max_ (×10^6^)	65.46 ± 1.88 ^b^	106.81 ± 1.13 ^a^	59.89 ± 2.79 ^c^
*λ*_max_ (nm)	338.8 ± 0.2 ^c^	344.5 ± 0.2 ^a^	340.3 ± 0.0 ^b^

The values with different superscripts in the same row are significantly different as per Duncan’s multiple range test (*p* ≤ 0.05).

**Table 4:** The mean particle size, ζ-potential, surface hydrophobicity (*H*_0_), intrinsic fluorescence intensity (*I*_max_), and maximum fluorescence emission wavelength (*λ*_max_) of the WPI aggregates formed through DPH pretreatment.

	Sample		
	P7T3	P7T5	P7T7
Mean particle diameter (nm)	178.1 ± 0.6 ^b^	3045.0 ± 2062.0 ^a^	178.1 ± 0.8 ^b^
ζ-potential (mV)	9.07 ± 0.89 ^a^	−5.82 ± 1.37 ^b^	−21.07 ± 1.55 ^c^
*H*_0_ (×10^6^)	114.40 ± 1.34 ^b^	233.34 ± 6.09 ^a^	20.30 ± 1.26 ^c^
*I*_max_ (×10^6^)	68.56 ± 0.80 ^b^	109.07 ± 4.98 ^a^	58.51 ± 0.83 ^c^
*λ*_max_ (nm)	339.0 ± 0.0 ^c^	344.3 ± 0.0 ^a^	340.2 ± 0.2 ^b^

The values with different superscripts in the same row are significantly different as per Duncan’s multiple range test (*p* ≤ 0.05).

**Table 5:** The mean particle size, ζ-potential, surface hydrophobicity (*H*_0_), intrinsic fluorescence intensity (*I*_max_), and maximum fluorescence emission wavelength (*λ*_max_) of the WPI aggregates formed through DPH post-treatment.

	Sample		
	T7P3	T7P5	T7P7
Mean particle diameter (nm)	169.1 ± 1.8 ^b^	2114.8 ± 115.6 ^a^	170.8 ± 1.9 ^b^
ζ-potential (mV)	9.14 ± 1.27 ^a^	−4.39 ± 0.85 ^b^	−19.41 ± 1.03 ^c^
*H*_0_ (×10^6^)	223.43 ± 2.28 ^a^	78.62 ± 1.92 ^b^	20.62 ± 0.12 ^c^
*I*_max_ (×10^6^)	64.82 ± 1.77 ^b^	70.73 ± 0.86 ^a^	59.62 ± 2.32 ^c^
*λ*_max_ (nm)	339.8 ± 0.2 ^a^	340.3 ± 0.5 ^a^	340.3 ± 0.5 ^a^

The values with different superscripts in the same row are significantly different as per Duncan’s multiple range test (*p* ≤ 0.05).

## Data Availability

Data is contained within the article.
